# Understanding Motivation, Career Planning, and Socio-Cultural Adaptation Difficulties as Determinants of Higher Education Institution Choice Decision by International Students in the Post-pandemic Era

**DOI:** 10.3389/fpsyg.2022.955234

**Published:** 2022-07-14

**Authors:** Kun Zuo

**Affiliations:** Shanghai Polytechnic University, Shanghai, China

**Keywords:** career planning, cultural adoption, higher education, personality development, innovative learning behavior

## Abstract

The world is facing an unprecedented health crisis with the spread of COVID-19 across different corners of the globe. This pandemic has raised more significant concerns about international students’ learning environment, personality development, and career planning, particularly in high-ranked institutes in China. Now the question concerning this dilemma is, would the COVID-19 pandemic negatively affect students’ education and the country culture where they are bound to seek information and the subject education? Therefore, this study examines the impact of innovative learning environment, career planning, and socio-cultural adaptation-related difficulties faced by international students as determinants of higher education institution choice decisions made by international students in the post-pandemic era. This quantitative study examined international students in high-ranked universities across China. The data from 260 students were collected through a structured questionnaire and analyzed using the AMOS technique. Moreover, it has been observed that the current global health crisis has intensified social inequalities across different international higher education systems. Countries fail to maintain the scale of the innovative international learning environment. The results further indicated that international students are more considerate of innovative learning environments, cultural adoption, career planning, and personality development, specifically after the outbreak of the COVID-19 pandemic, which has drastically affected the global higher education system. Unusually, more than half of the participants wanted to maintain the option of overall distance education after the pandemic. However, apart from this argument, it is appropriate to demand significant changes in post-pandemic education adapted to the post-digital era and to satisfy the concerns and expectations of the students.

## Introduction

The emergence of the COVID-19 pandemic in early 2020 has unleashed havoc of its kind on the entire world, affecting almost all aspects of human lives; the higher education system was no different. Primarily, it caused delays and postponement of many physical events and activities, which resulted in a rapid shift of physical teaching to the digital one, leading to the emergence of “New Normality” in the higher education system (Tesar, 2020). This emergence of COVID-19 has brought new opportunities in research collaboration, education, learning, and institutional governance. The higher educational institutes were made to re-think and re-design their overall structure for the modern and dynamic execution of the knowledge dissemination process. With the implementation of immediate changes, such institutes require contingency initiatives and risk-mitigation strategies to continue growing innovatively. Even though this pandemic has forced the higher education institutes to re-form the role of the innovative learning environment through digitalization and to educate, formulate, and induce those innovative skills in the students, which can bring international students to the next level of learning.

Besides, the prevailing pandemic has adversely affected the student’s personalities because of their physical and technological exposure to the educational institute, and its socio-cultural learning and adaptions. Moreover, the given study highlights the hidden aspects associated with international students’ personality development constraints. It also opens the additional avenues for the higher education systems to re-think and re-consider the personality development aspects of the students, which are the crucial aspects in their career planning, and also in the formation of university image and ranking. Recently, there has been a rising interest in international education, as many students travel abroad to pursue higher education in developed nations (Scotland, 2016). Several reasons attract students toward international higher education, including international education, as an essential source of their competitive personality grooming. They were followed by providing improved chances of skills with the help of technology-based educational facilities.

Along with all the positives, higher education confronts several challenges in its journey toward adopting emerging learning models and meeting dynamic needs (Scotland, 2016), including issues related to educational technology and cultural and socio-political adoption, specifically in the post-COVID era (Murray and Lang, 1997). It is essential to be mindful that advanced-level teaching and learning cannot be translated into a reality without the influx of technology. Specifically, it is essential to nurture the skills demanded by the job market in the 21st century (Liesa-Orús et al., 2020).

It is obvious that the people travel to different countries and are more interested in learning the other countries’ cultural norms (Ghani et al., 2020). The government is taking different initiatives to ensure that the students are provided with every kind of facility in their international high-ranked institutes. They must not be discriminated against because of their varied cultural affiliation (Rae, 2007). Similarly, it is also the responsibility of the people of the host country to be more tolerant toward international students and let them embed themselves into the host nation’s culture. Furthermore, Aboramadan (2020) suggests that it is also the responsibility of the international students to promptly integrate with the international culture and opt for strategies to develop their academic profile effectively. Likewise, career planning is supposed to be one of the primary motives of every international student, which is why they prefer developing their academic profiles for a better career (Nghia et al., 2019; Taylor et al., 2019). The urge for a brilliant professional career forces most aspirants to travel abroad for quality education.

Similarly, through innovative quality academic learning, international students reasonably develop their personalities for a better professional career (Pan et al., 2021; Chuang et al., 2022). It is believed that those students who get an education in the international institutes of a high standard have more robust and influential personalities than those who do not have the opportunity to learn in the international institutes (Jackson and Tomlinson, 2019). In developed countries, cultural difference is one of the main problems for the international students. It is essential to understand that the students with high cultural adoptive profiles sail through all the technological and socio-economic-political hiccups. However, the students with a dominant mother culture personality face different problems in the way of their cultural adoption (Scotland, 2016; Blynova et al., 2020).

Furthermore, it has been observed that there is a definite shift in international academic institute structure, which has given rise to the unique, innovative educational possibilities to diversify teaching and learning procedures (Imms et al., 2017; Mackey et al., 2018). As such international learning environments have emerged at a rapid pace, they have been termed the innovative learning environments, which hold the potential to engage, motivate, and attract a more significant number of international academic aspirants (Oced, 2015). It is one of the reasons why the desire to be part of institutes with innovative learning environments is quite prevalent among students interested in pursuing higher education. Moreover, according to Ige et al. (2020), innovative learning behavior is one of the key traits that help people develop their profile by identifying new ways of learning. Students with innovative learning behavior are more substantial and effectively learn things (Chou et al., 2019). However, to reach its full potential, it is essential to realize that digital evolution across international higher education has made all stakeholders undergo identifiable changes because of this innovative learning shift. It requires a sufficient adaptation on behalf of students, teachers, and the international institute at large (Mahlow and Hediger, 2019; Abad-Segura et al., 2020).

Consequently, to test the phenomena at hand, this study uses the prism of higher education theory. Therefore, this study aims to understand the role of an innovative learning environment, career planning and socio-cultural adaptation, and difficulties as determinants of higher education institution choice decisions by international students in the post-pandemic era. In pursuit of testing the above-stated relationships, it is imperative to understand that the cultural and social botherations caused by the host countries are some of the primary roadblocks to the success of the international academic institutes. These indicators hinder international students from seeking admission to such institutes (Gurin et al., 2002; Scotland, 2016; Bennett, 2019). Therefore, this study is designed to understand to what extent of innovative learning environments, career planning, and personality development opportunities lead international students to get an education in the international institutes in the post-pandemic era. Significantly, the theoretical framework of this study has been extracted from recent studies with inconclusive findings. Notably, past studies lack understanding of innovative learning environment, career planning, and socio-cultural adaptation difficulties as determinants of higher education institution choice decisions by international students in the post-pandemic era. Therefore, the framework of this study is designed to provide meaningful information related to the motivation, career planning, and cultural adaptation difficulties in the context of the higher education of the international students in developed and advanced countries.

To the researcher, no particular study was conducted to address the relationship between career planning, personality development, cultural adoption, and higher education in international institutes. However, the theoretical implications of this study would address the gap in the literature and emphasize the role of other variables that bother international academic aspirants and institutes interested in having such students on board. Similarly, the practical implications of this study would be helpful for the stakeholders to consider different variables used in the study for managing and improving the behavior and thinking of the international students to integrate with the culture of the host country’s people. Furthermore, this study provides significant future directions that are important for future researchers not to repeat the same work but go with an alternative strategy.

## Literature Review and Hypothesis Development

**FIGURE 1:**
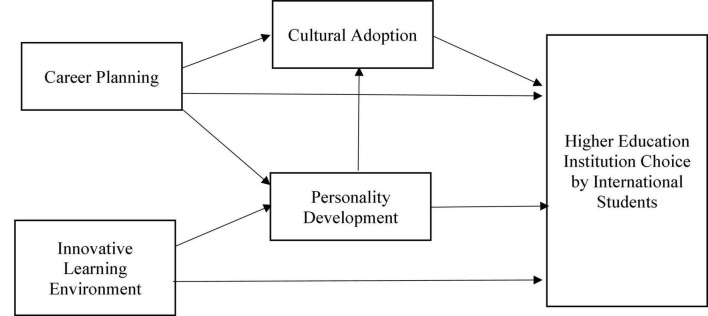
Research model.

In this study, the research model is designed on the interpretation of the theory of higher education (see Figure 1). The theory of higher education highlights that the students are more concerned and affected by their thought patterns and beliefs, which they establish over time (Gurin et al., 2002). Therefore, this theory emphasizes higher education management’s importance in considering these values and providing a sustainable working environment for the students to learn (Chan, 2004). Furthermore, the interpretive lens demonstrates that by providing a conducive environment for academic growth, college students will be in a better position to perform in a productive manner (Maringe and Gibbs, 2008). However, during the literature review, it was identified that multiple other factors contribute to the sustainable development of higher education for college students. This study has considered multiple other variables such as innovative learning environment, personality development, career planning, and cultural adoption to understand motivation, career planning, and socio-cultural adaptation difficulties as determinants of higher education institution choice decisions by international students in the post-pandemic era. Also, based on these additions to the body of knowledge, stakeholders across the higher education system would be in a better position to emphasize factors that are more critical for students interested in pursuing international higher education in the post-pandemic era.

### Relationship Between Career Planning, Cultural Adoption, and Personality Development

It is essential to understand that carrier planning is one of the prime objectives of international students when they are willing to get admission to international institutes for their higher education (Scotland, 2016). It is also observed that the students of third-world countries are more interested in getting admission across developed nations to groom their personalities (Murray and Lang, 1997; Scotland, 2016). However, according to Martín-García et al. (2019), it is not easy to get admission to an international institute because most institutes are backed by several cultural, political, and social norms, and rules and regulations. Studies have revealed that international students face cultural crises during their academic stay at international educational institutes (Gurin et al., 2002; Johnson et al., 2016). International students will likely experience difficulties adapting to their new context (Gong et al., 2021).

It is hard for the international students to survive in the international institute because, in such kinds of institutes, different cultural and social dimensions challenge the ethical norms, and it is not easy to integrate with the student of other cultures (Ghasemian Safaei and Farajzadegan, 2012; Liu et al., 2021; Zhong et al., 2021; Sulistyarini et al., 2022). As such, integration might demand multi-level (i.e., psychological and social adaptation) accommodation (Berry, 2005); therefore, it would be fitting to expect that international students would have to go through a challenging, life-changing encounter. Conversely, in America, according to the study by Thaothampitak and Wongsuwatt (2022), the students of different countries are getting an education in the higher institutes, and it is believed that American institutions are suitable for personality development and career development.

Besides, it is also essential to consider that international students in American institutes are not treated well because of socio-cultural evil that disturbs the educational process across institutes (McGettigan and McKendree, 2015; Odinokaya et al., 2019; Sormunen et al., 2020; Zhong et al., 2021). Similarly, in Canada, students from different countries are getting an education; however, the cultural variation and different social backgrounds are hurdles in developing their personalities, with no counter strategies available for the issue at hand (Hadziabdic et al., 2016; Zhong et al., 2021; Sulistyarini et al., 2022). Likewise, it is critical to understand that it is not easy for people to accept others from different cultural backgrounds. While, on the contrary, such international institutes have a reasonable extent of cultural diversity among students. Moreover, people from different cultures pursue their academic endeavors together, so it is also the responsibility of the institute’s management to apply policies to avoid cultural clashes (Scotland, 2016; Saddhono, 2018; Al-Jubari, 2019; Kazemitabar and Garcia, 2021).

In the international institutions of Korea, it is reported that the domestic students are more reluctant toward the international students, and most are biased. They do not treat the international students according to their culture and ethical values (Chan, 2004). It has also been observed that institutes across Korea arrange cultural classes for students who apply from the third world and Asian countries, to be specific, which is adding to the reluctance of international students to consider Korea for their international academic endeavors (Nghia et al., 2019; Taylor et al., 2019). Furthermore, international students willing to be admitted to the international institutes are always interested in the host country’s culture. The students believe that the cultural class of the first country would not allow these people to get an education in the right way and would not help them develop their personalities effectively (Gaunt and Treacy, 2020; Hunter and Frawley, 2022). In this regard, the responsibility of the educational institute is to determine the different kinds of problems that are hurdles in the way of admission of the international students to provide the solution for them that would be refractive for their learning (Nghia et al., 2019; Barlett et al., 2021; Zhong et al., 2021). Most of the time, international students face a different kind of crises in international education. Due to such a crisis, their personality development declines, becoming one of the prime reasons behind negative word of mouth.

Importantly, in the era of the pandemic, it is noted that students studying in international institutes have faced different kinds of cultural problems. In the American universities, the Chinese student was blamed for the COVID-19 virus, which supposedly happened because of a political narrative set by one nation against the other (Javaid et al., 2016; Chotimah et al., 2021; Zhong et al., 2021). On the contrary, it is also essential to understand that the people who represent the different cultures must believe that they should treat others ethically. Importantly, it is the government’s responsibility to provide ethical education to the people and develop students effectively for their better personalities and understanding (Aboramadan, 2020; Zhong et al., 2021). Therefore, it is crucial to understand that those international institutes that provide students with proper training and career planning and counseling will be in a better position to make them adopt the host culture.

H1. There is a relationship between career planning and cultural adoption.

H2. There is a relationship between career planning and personality development.

H3. There is a relationship between career planning and higher education.

### Relationship Between Innovative Learning Behavior, Personality Development, and Higher Education

People from multiple cultural backgrounds live together in international academic institutes, representing their cultures and seeking education. Besides, it is a trend that international students are getting admission into higher education because they believe that getting higher education from a reputable international institute would develop their personality and career through innovative learning practices (Chin et al., 2019; Taylor et al., 2019). Further, people from different cultural backgrounds *seek education from* international institutes and *become* more concerned about the host country’s culture and political system, contributing a lot to their personality development and learning (Elumalai et al., 2021; Prewett and Whitney, 2021; Zhong et al., 2021). In this regard, it is not only the responsibility of the people of the host country to value and respect the international students but also the responsibility of the people who are getting admission to the international institutes to value the innovative learning practices, culture, and the social-economic system of the host country (Al-Jubari, 2019; Sulistyarini et al., 2022), while failure to adjust to the technology-based creative atmosphere can be subjected to the apparent disconnect between students’ culture, host institutes’ culture, and their leadership. Therefore, it is believed that the host nations’ mutual trust and mutual respect, and academic and national cultural values of each other help develop better harmony among locals and international students. Importantly, with the help of globalization and different drivers of globalism, it has become easier for the people to understand the culture of the people as trade and technology have ensured easy cultural diffusion (Palaschuk et al., 2022).

It has helped locals realize the importance of accepting the guest students’ cultural differences and adjusting themselves accordingly. In addition, innovative learning behavior is crucial for an average student to adjust to the international education scenario, as it helps them accept the emerging innovative academic and cultural dimensions, which make them acceptably alter their personalities (Saykili, 2019; Ige et al., 2020; Hoseini et al., 2022). Likewise, to make this procedural and psychological adjustment by students easier, critical analysis on how such an influx of innovative learning models will benefit students’ academic outcomes is unavoidable. Similarly, unquestioned implementation of educational innovations might hinder innovative learning procedural adoption (Castañeda and Selwyn, 2018). In addition, in their study (Byers et al., 2018), have discussed the impact of an innovative learning environment on students’ behavioral outcomes. They have referred to an educational environment as a “blended learning environment,” with a sufficient level of digitalized learning facilities. It was also found that there has been an improvement in students’ learning and behavioral outcomes once they get a chance to adapt and adjust in such a blended learning environment.

That is why the students in international institutes are more developed in their studies than those who get an education from a domestic institute (Asad et al., 2020). In America and the United Kingdom, the educational institutes are providing the opportunity for the international student to get the higher education from the facilitated and well-established institutes. The research and development have been established effectively and creatively, contributing to the students’ personality (Saykili, 2019; Pan et al., 2021; Zhong et al., 2021). This way, the trend of getting an international higher education has increased over the past three decades. The students are more interested in getting an education in the international institutes because they are well-informed about the clear distinction between the students of the national and international institutes (Gurin et al., 2002; Scotland, 2016; Catarino et al., 2019).

Notably, the students are always willing to develop their personality because they believe personality is constantly changing concepts. With the help of more innovative learning behavior, it would be appropriate for the students to learn things critically (Saykili, 2019). Still, a good contradiction exists on how a technology-based innovative learning environment can help enhance student learning and engagement. Therefore, there is a need for a well-thought influx of innovative learning tools, especially the speedy digitalization of the education system during the COVID-19 pandemic. It has made it imperative for the international students to stay ahead in terms of being flexible to cultural, technological, and social variations they will face (Fletcher et al., 2020). Therefore, it is hypothesized as follows:

H4. There is a relationship between an innovative learning environment and personality development.

H5. There is a relationship between an innovative learning environment and higher education.

H6. There is a relationship between personality development and higher education.

### Personality Development, Cultural Adoption, and Higher Education

Personality is another critical component of human behavior and action. It is a fact that every individual has a different set of personality traits, and he is entirely different from the other human being. Personality is one of the subjective approaches to human behavior that helps to grow and provides more of a gift for improving the behavior (Pan et al., 2021; Chuang et al., 2022). In the career development of any individual, the role of personality is critical because it matters a lot when one is the applicant for getting any job (Sabater-Grande et al., 2022). In this way, the students are more conscious of developing their personality and attitude to the advanced level for better jobs in the industrial sector. Significantly, Bhatti et al. (2018) noticed that the third-world countries are not appropriately working to improve the students’ personalities to the required extent. In this regard, the students of the third-world countries are more interested in traveling abroad and getting an education from an international standard academic institute to develop their personality appropriately (Ghufron and Ermawati, 2018; Cleary et al., 2022; Peng et al., 2022). In the past, the tradition of traveling for international education was not common because different cultural and social barriers inhibited the people from getting an education from the international institute for higher education (Peng et al., 2022).

However, with the development of globalism and global institutes, it has become more accessible for people to get admission to international institutes for education. In this way, the students from different third-world countries and the Asian countries travel to the developed and advanced countries to get an education and develop their personalities according to the modern standard (He and Chiang, 2016). It is critical to understand that if the students are allowed admission to the international institutes, their personalities will be shaped according to the international employer requirements (Peterson, 2001; Ghasemian Safaei and Farajzadegan, 2012). It indicates that personality development is one of the critical criteria for shortlisting the academic institute held by students interested in applying to international higher education institutes. Therefore, the international institutes that provide students with facilities to groom their personality as demanded by reputed employers will indeed be preferred by potential students for their higher education. The reputed organization will credit the intellectual personalities because of their personal and professional interactions (Pan et al., 2021). Knowing that other people attract people with good personalities and positive attitudes is critical because they are more positive and do not react to their perceived values and norms.

However, on the contrary, people with low personalities and the capability to work with high standards are not self-motivated. They are limited to their personality as they are not getting the opportunity for the best education. In this way, the students from Pakistan, India, and China travel to the international institutes and high-class universities in America and the United Kingdom to seek education and enhance their skills related to research and development (Wilkinson et al., 1998; Asmus, 2021; Cleary et al., 2022). This research and developments are not only necessary for the students for the development of their personality, but also it is critical for the people of different countries because, with the help of these personality characteristics, people are more advanced to get the maximum benefit from the society and the educational institutes. Importantly, it is the responsibility of the stakeholder to consider personality as a unique and distinctive human characteristic and provide the opportunity to boost it to the students and other people of the society (Bhatti et al., 2018; Aguillon et al., 2020; Yusup et al., 2022). If the opportunity was provided to the people to enhance their personality skills, it would be beneficial for them to get the right skills and develop more strategies for maximum benefit. It also indicates that in developing one’s personality, the student willingly agrees to adapt to the culture, which is not the primary culture. Students work to adapt academic, professional, and socially benchmarked cultures to achieve the desired performance outcomes. Therefore, according to Pan et al. (2021), it is also the responsibility of the international institutes’ management and board of directors to integrate personality with human life and provide opportunities to the international student to develop their personality to the appropriate level. It is also noted that if the opportunities related to personality development are provided to the students in the international institutes of higher education, the development of human skills would be enhanced, and people would become more competent in their respective fields (Onan, 2021; Regier, 2021; Kurniawan and Andani, 2022).

Likewise, the students from Japan are more concerned about their personality development when they are getting an education in the educational institutes of western countries. Similarly, the English students in America and the United Kingdom are working to develop their personalities and critical skills with the help of higher education because they have the opportunity to get an education in the well-reputed and well-established institutes in the world (Odinokaya et al., 2019; Kurniawan and Andani, 2022). In the same manner, the students from every country must be provided with the opportunity for personality development by the management of the educational institute to grow productively to the advanced level for a more efficient result (Odinokaya et al., 2019; Nasir and Dermawan, 2022). Therefore, it is proposed as follows:

H7. There is a relationship between personality development and cultural adoption.

H8. There is a relationship between cultural adoption and higher education.

## Methodology

### Prepare Questionnaire

This study is based on the quantitative data the target respondents collected *through the* survey-based method. It is noted that the survey-based data collection method is the appropriate method as it is time-saving and cost-saving. In this regard, the *structured* questionnaire was prepared on a five-point Likert scale to collect the data to determine the relationship between different variables presented in the study’s theoretical framework. Notably, the scale items in this questionnaire were taken from the creditable earlier studies. The scale items for personality development were taken from the study of Penketh et al. (2014). Similarly, the scale item of innovative learning environment was taken from the study of Rosedi et al. (2015).

Moreover, the scale items for cultural adoption were adopted from Hadziabdic et al.’s study, the scale items for higher education were adopted from Scotland (2016), and the scale items for career planning were taken from the study of Presti et al. (2013). In addition, these scale items were reviewed by expert researchers to check their significance and worth for the study.

### Data Collection Process

The questionnaire of this study was developed carefully and ensured that the integrated scale items were essential and reliable for collecting the data from the respondents. The respondents of this study were the students of different countries that are getting admissions and getting an education *in international institutes across China*. Moreover, the data were collected from students of international institutes, and 600 questionnaires were distributed to the respondents in China. In this way, the study’s introduction also provided a better understanding of the subject. Similarly, the paid envelopes were also provided to them for the return of the questionnaires. The respondents were welcome to ask any question, and in this regard, they were provided with the researcher’s email to get an answer related to the questionnaires. However, the incorrect questionnaires were not considered for the study. Finally, 260 questionnaires were considered for the study to analyze the data for the study’s hypotheses.

## Findings

This study section has the confirmatory factor analysis results which are presented in Table 1. Confirmatory factor analysis is conducted to determine the values of factor loadings for the scale items of variables used in the study. Significantly, the values of factor loadings for all scale items were not less than 0.40, as recommended by Hair et al. (2017). Furthermore, the scale items used to measure the data are presented in this study section. These scale items were carefully considered to determine the relationship between the hypotheses developed based on the theoretical framework of this study.

**TABLE 1:** Results of confirmatory factor analysis.

Variable	Items		Factor loadings
Career planning	CP3	Determine the steps you need to take successfully complete your chosen major	0.885
	CP2	Prepare a good resume	0.558
	CP1	Make a plan for your goals for the next 5 years	0.592
Innovative learning environment	ILE3	I benefit more from the computer lab class	0.836
	ILE2	I prefer to learn by doing exercises	0.869
	ILE1	When I do things in class, I learn better	0.775
Cultural adoption	CA4	I am less patient with individuals of certain cultural backgrounds	0.893
	CA3	I often react to how culture affects beliefs, attitudes, and behaviors	0.556
	CA2	I think my behavior is influenced by my culture	0.563
	CA1	I think my beliefs and attitude are influenced by my culture	0.520
	HE4	Everyone in the group will have an equal opportunity to participate	0.932
	HE3	It is fair that everyone in the group gets the same marks	0.946
	HE2	Everyone in the group will do an equal amount of work	0.858
Higher education	HE1	I will get a higher grade working in a group than working individually	0.420
	PD3	The individuals like to be given a lot of directions	0.882
	PD2	The individuals don’t listen to the rules	0.886
Personality development	PD1	The individuals accept imposed limits	0.883

### Measurement Model

**FIGURE 2:**
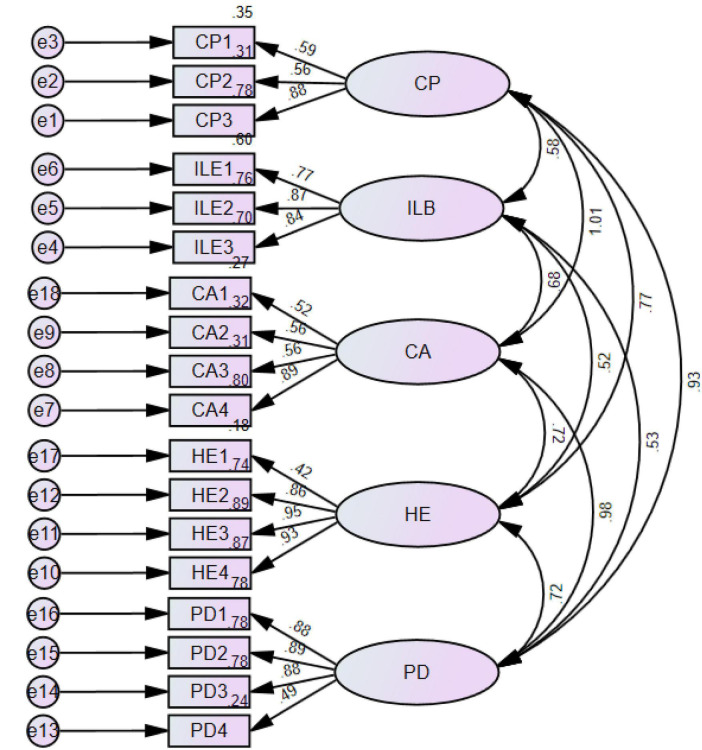
Measurement model. CP, cultural performance; ILB, innovative learning behavior; CA, cultural adoption; HE, higher education; PD, personality development.

In this study section, AMOS software was used to determine the reliability and validity of the scale items used (see Figure 2). The results reveal a clear correlation, reliability, and validity (see Table 2). Furthermore, the composite reliability (CR) values were not less than 0.70, as recommended by Henseler et al. (2014). Also, the average variance extracted (AVE) value was not less than 0.60 in this study, as recommended by Henseler and Fassott (2010). According to the results, there is apparent reliability and validity between the scale items used for each study variable.

**TABLE 2:** Reliability, validity statics, and correlations.

	CR	AVE	MSV	MaxR (H)	CP	ILB	CA	HE	PD
CP	0.727	0.682	0.523	0.821	0.894				
ILB	0.867	0.685	0.568	0.873	0.583***	**0.827**			
CA	0.736	0.624	0.523	0.840	1.011***	0.684***	**0.766**		
HE	0.883	0.669	0.592	0.948	0.770***	0.523***	0.717***	**0.818**	
PD	0.875	0.697	0.551	0.917	0.925***	0.532***	0.975***	0.721***	**0.831**

CP, cultural performance; ILB, innovative learning behavior; CA, cultural adoption; HE, higher education; PD, personality development. The bold values indicate the results for corresponding statistics for whole variable. ***Significant at 1%.

In addition, the measurement model fit was determined by analyzing and evaluating the root mean square of approximation, absolute fit measures, standardized root mean square residual, comparative fit index, normed fit index, and adjusted goodness of fit (see Table 3). Importantly, all the values were appropriate for the recommended threshold for it.

**TABLE 3:** CFA model.

Measure	Recommended threshold	Abbr.	Scores
Chi-square/df (CMIN/DF)	<3.0	2/df	2.0021
Comparative Fit Index	>0.90	CFI	0.7
The Normed Fit Index	>0.90	NFI	0.79
Goodness of fit	>0.90	GFI	0.86
Adjusted Goodness of fit	>0.80	AGFI	0.78
Root Mean Square Residual	<0.08	RMR	0.09
Standardized Root Mean Square Residual	<0.08	SRMR	0.09
Root Mean-Square Error of Approximation	<0.08	RMSEA	0.09

### Discriminant Validity

The discriminant validity was checked using heterotrait–monotrait (HTMT) method to understand the distinction used for each construct of every variable. Significantly, the results reveal that all the values of discriminant validity were not less than 0.90, which Hair et al. (2017) recommended for modern studies. Therefore, according to the results, the constructs used for each study variable have apparent discriminant validity.

### Structural Model

In this section of the study, the results of the hypotheses tests are presented in Table 4. H1 was tested to check its significance, and according to the results, career planning has a significant effect on cultural adoption (β = 0.311, *t* = 3.821, *p* = 0.000), and H1 is accepted. H2 was tested to check its significance, and according to the results, career planning has a significant effect on personality development (β = 0.322, *t* = 3.527, *p* = 0.000), and H2 is accepted. H3 was tested to check its significance, and according to the results, career planning significantly affects higher education (β = 0.315, *t* = 3.871, *p* = 0.000), and H3 is accepted. H4 was tested to check its significance, and according to the results, an innovative learning environment significantly affects personality development (β = 0.367, *t* = 3.782, *p* = 0.000), and H4 is accepted. H5 was tested to check its significance, and according to the results, an innovative learning environment significantly affects higher education (β = 0.351, *t* = 4.891, *p* = 0.000), and H5 is accepted. H6 was tested to check its significance, and according to the results, personality development has a significant effect on higher education (β = 0.359, *t* = 3.701, *p* = 0.000), and H6 is accepted. H7 was tested to check its significance, and according to the results, personality development has a significant effect on cultural adoption (β = 0.353, *t* = 4.112, *p* = 0.000), and H7 is accepted. Lastly, H8 was tested to check its significance, and according to the results, cultural adoption significantly affects higher education (β = 0.214, *t* = 3.647, *p* = 0.000), and H8 is accepted.

**TABLE 4:** Discriminant validity—HTMT.

	CP	ILB	CA	HE	PD
CP					
ILB	0.610				
CA	0.849	0.772			
HE	0.860	0.657	0.826		
PD	0.870	0.578	0.853	0.835	

*CP, cultural performance; ILB, innovative learning behavior; CA, cultural adoption; HE, higher education; PD, personality development.*

## Discussion

Table 5 shows the Standardized path coefficient with H1,2,3,4,5,6,7,8. The findings of H1, H2, and H3 demonstrate a significant relationship between career planning, cultural adoption, personality development, and higher education. It is essential to understand that international students travel to first-world countries to get higher education because they want to develop their careers successfully. Indeed, according to Scotland (2016), it is suitable for students who are willing to develop their personality and career by getting an education from a world-class university. However, different barriers, such as cultural adoption and social barriers, must be eliminated. In this way, the opportunities should be provided to the students willing to get an education from the international institutes (Hina et al., 2019; Hoseini et al., 2022). It is the responsibility of the government and the peacemaker non-government organizations to intervene and modify the cultural awareness of the students and provide them with information to tolerate the culture of the host country (Pan et al., 2021).

**TABLE 5:** Standardized path coefficient.

Hypotheses	Relationship	Beta	*t*-values	*p*-values	n
H1	Direct	0.311	3.821	0.000	Accepted
H2	Direct	0.322	3.527	0.000	Accepted
H3	Direct	0.315	3.871	0.000	Accepted
H4	Direct	0.367	3.782	0.000	Accepted
H5	Direct	0.351	4.891	0.000	Accepted
H6	Direct	0.359	3.701	0.000	Accepted
H7	Direct	0.353	4.112	0.000	Accepted
H8	Direct	0.214	3.647	0.000	Accepted

Moreover, it is also understood that for the development of culture and enhancement of regulations related to the culture, more information must be provided to the people to develop them in a productive way for tolerating the culture and the people associated with it. The Chinese students who are getting an education in the universities in the United States are more aware of the cultural clashes, but they tolerate kindness for their more significant benefit (Chan, 2004; Tsai et al., 2020). Similarly, cultural awareness of this kind must be provided to the student of the other countries to ensure that they are not rejected. However, they must consider and value the culture of the other community (Hina et al., 2019). In this way, creating unity in the cultural understanding would be an excellent opportunity for developing a mutual relationship between the community for progress and productivity.

The results of H4 and H5 demonstrate a significant relationship between an innovative learning environment, personality development, and higher education. However, it is noted that the innovative learning environment is critical for international students’ learning when they are getting an education in world-class universities. The students of Japan who are getting an education in Australia and the United Kingdom believe that if they are provided with a sustainable and comfortable environment of learning with the security of innovative learning behavior, then it would be more effective for them to develop their ability constructively (Scotland, 2016; Pan et al., 2021). The international institutes are responsible for providing opportunities to international students to enhance innovative capabilities and personality development (Martín-García et al., 2019). Besides, it is reasonable to understand that the purpose of every student is to get an education from foreign institutes for the development of the personality because this development in personality provides the opportunity for better understanding (Ntobuo et al., 2018; Saykili, 2019). Significantly, the appropriate actions must be taken to create an innovative learning environment with the help of effective and innovative teaching methods to improve the student’s performance to the advanced level. Notably, more innovation in the learning *must* be provided to the students as a result. It solves the problem (Watson, 2007; Hou et al., 2021). In this manner, the higher institutes must be responsible for developing innovative learning strategies and improving the students’ performance.

The results of H6 demonstrate a significant relationship between personality development and higher education. Indeed, the critical motive of any student is to get higher education for the personality development, because until and unless any institution is not providing the opportunity for the personality development of the student, then it would not be reliable for the student to get admission in that institution (Carvalho and West, 2011; Ghufron and Ermawati, 2018). Besides, the international institutes hosting international students are more flexible in providing the opportunity for personality development to the students because by providing these opportunities, the institutes are attracting the students (Saykili, 2019; Hoseini et al., 2022). The institutes contributing to the student’s personality are more considerable by the international students for the higher education. The results of H7 demonstrate a significant relationship between personality development and cultural adoption.

Furthermore, the results of H8 demonstrate a significant relationship between cultural adoption and higher education. In this regard, it is noted that the students willing to get admission to the international institute for higher education are more concerned about their personality development and profile status. Moreover, some students believe that the host country’s culture would be challenging for them in their higher education as the people of different cultures are not ready to tolerate the values of the people of the other culture (Aboramadan, 2020; Ghani et al., 2020). Importantly, it is the responsibility of the government and international university management to provide suitable facilities to the students for the development of their personality and socio-culture adaptation exposure (Zhu et al., 2010; Elumalai et al., 2021). Similarly, the understanding of the culture and emotional intelligence must be provided to the society, so the people should not be face to face for the concerned about their cultural values (Bayanova et al., 2019; Veissière et al., 2020; Mashudi et al., 2022). In addition, it is noted that in the era of globalism, everyone lives in one integrated culture, but the traditional concept of cultures is not eliminated (Kember et al., 2000; Oleksiuk and Rebrova, 2018; Saykili, 2019). Therefore, the management must provide reasonable facilities for cultural tolerance to boost the personality of the international students.

## Conclusion

This study concludes that there is a significant role of personality development, career planning, innovative learning environment, and cultural adoption provided by the higher educational institute in attracting more international students to take admissions. The study indicates that international higher education institutes need to be mindful of the emerging student’s requirements and their rising concerns in the post-COVID-19 era. Indeed, international students are getting an education in America and Canada because they believe their profile would be developed if they get an education from high-class institutes. They would learn critical abilities to understand the problem and cope with the problems in an effective way. Therefore, the implications of the study would be significant for the stakeholders to provide the best and most innovative learning environment by eliminating all of the barriers in the way of international students who are getting admission to the world-class universities of the top world countries. Importantly, this study demonstrates that the gap in the literature was identified that had been addressed by developing the research framework that would be useful for future studies to understand the relationship between different variables that are taken to develop the hypotheses of the study. In addition, the study provides significant future directions that are important to consider for future researchers interested in pursuing research in the given domain in the post-COVID times. Moreover, this study highlights that the responsibility of the international students is to integrate with the host country’s culture and develop their personality in the innovative learning environment for career planning by getting a higher education.

## Implications

### Theoretical Implications

This study is designed to provide significant theoretical implications related to the admission and education of international students in the well-reputed international institutes of developed countries. It is a fact that every student wants to develop his personality and career by getting an education from credible international institutes because the educational institutes contribute a lot to the development of a file of the students. In this regard, it is crucial to understand that there are different problems related to the culture and tradition of the international students that limit them from participating in activities to getting an education from the international institutes. However, very few studies were conducted to determine the relationship between different cultural factors in getting admission to international institutions in the post-pandemic era. In this way, the study highlights that the students who are willing to get an education from the international institutes must consider the vital role of personality development and career planning because the educational institutes matter a lot while getting a job in the industry.

Similarly, the study demonstrates an essential role of cultural adoption in international education because it was not considered by any earliest studies to influence the students’ personality in the international institutions for higher education. Importantly, this study highlights that the innovative learning environment is also critical for the students to be understood for getting an education in the international institutes. In this manner, the study provides the relationship between different variables that are taken in the research framework of the study that would be useful for future researchers to determine the relationship between variables related to the higher education of the students in the international institute.

### Practical and Managerial Implications

This study also provides significant practical implications that are important to consider for the stakeholders providing international higher education to the students traveling from different countries to pursue their educational degrees. It is essential to understand that the international students are traveling to the developed countries for an education because they believe that getting an education from the developed countries would be beneficial for them to develop their personality and skills for working and getting a job in the reputable departments. However, it is reasonable to note that there are different kinds of challenges posed by increasing competition and the COVID-19 pandemic that international students face in the international scenario because they belong to different cultures. In this way, it is the government’s responsibility and the management of the higher institute to regulate human behavior and provide awareness related to the importance of patience required by international students. Notably, the students are traveling to the developed countries to get an education and want to settle there and get professional life in the developed countries. In this way, it is the responsibility of the host country’s government to integrate the international students with the local students effectively to provide the best facilities for learning from each other. Moreover, it is the government’s responsibility to ensure that the students are not discriminated against based on culture and must be given equal opportunities to get better results for their living standards. In addition, it is the student’s responsibility to value the beliefs and norms of the opponent culture. Because if there is no emotional intelligence to determine the cultural changes, it would be difficult for the international students to survive and pursue their education at international universities.

## Limitations and Future Directions

This study aimed to understand motivation, career planning, and socio-cultural adaptation difficulties as determinants of higher education institution choice decisions by international students in the post-pandemic era. Like any other study, the given one is also faced with certain limitations, which can be the source of future direction for other research. Initially, due to time constraints, the study deals with a cross-sectional research design. However, the phenomena can be studied better if data could be collected from students who aspire to get admission to international academic institutes and when they end up with their degree programs. Therefore, the longitudinal research design is suggested for future studies in the given work area.

Similarly, the data were collected only from international top-ranked institutes from China, so there can be an issue of generalizability. Therefore, it is suggested that future studies could look into the phenomena from a comparative viewpoint. The literature review observed that multiple other factors contribute to the motivation and career planning for higher education in international institutes. First, future studies must consider the important role of visa policy in getting admission to international institutes. Second, future studies must focus on ethical values’ role in international students’ problems. Lastly, future studies should focus on government policies’ role in taking admission into international institutes, specifically in the post-pandemic era.

## Data Availability Statement

The original contributions presented in this study are included in the article/supplementary material, further inquiries can be directed to the corresponding author.

## Ethics Statement

The studies involving human participants were reviewed and approved by the Shanghai Polytechnic University, China. The patients/participants provided their written informed consent to participate in this study. This study was conducted following the Declaration of Helsinki.

## Author Contributions

KZ conceived and designed the concept, collected the data, wrote the manuscript, and read and agreed to the published version of the manuscript.

## Conflict of Interest

The author declares that the research was conducted in the absence of any commercial or financial relationships that could be construed as a potential conflict of interest.

## Publisher’s Note

All claims expressed in this article are solely those of the authors and do not necessarily represent those of their affiliated organizations, or those of the publisher, the editors and the reviewers. Any product that may be evaluated in this article, or claim that may be made by its manufacturer, is not guaranteed or endorsed by the publisher.
